# A Hospital Secretary's View

**Published:** 1920-07-24

**Authors:** 


*To the Editor of* The Hospital.

### A HOSPITAL SECRETARY'S VIEW.

*To the Editor of* The Hospital.

Sir,—While cordially endorsing the recognition which The Hospital has given to the De La Rue scheme, like the critic in the article headed as above, I have a "Yet."

"If there is one principle on which the profession agree it is that the patient enjoys free choice in the selection of his adviser."

In consultative practices this appertains, and rightly so; but is the scheme propounded by Messrs. De La Rue & Co. on the same basis? To me it savours of a half-way house between the patient who can afford to choose and the one who is placed in a cruel dilemma in having to accept charity because of the absence of the wherewithal to choose. This patient has two alternatives—the first to accept charity, which is repellant to him; and the second to cripple his finances by trying to meet the fees of a specialist and possibly subsequent nursing home charges. The second alternative is beyond the means of the class of employee for whom the firm desires to provide, for the originators of the scheme know exactly the amount of income earned by each patient, and it is as an alternative to the enforced—although unwilling—entry into a charitable institution that the firm—at least, so it appears to me—have initiated the scheme.

Take the case of a patient under his own medical adviser. He is progressing none too favourably, and the medical man, being dissatisfied, suggests a consultation. It is the general practitioner who usually chooses the specialist, and does so with a fair knowledge of his patient's ability to meet the fees. The patient certainly has the right to choose Sir Wm. Scalpel, or even Lord Bistoury, but what purpose does it serve, even with the

**The Editor's Letter Box—(*continued*).**

right to choose, if the patient's pocket is insufficient? Is not this a parallel of the difficulty the De La Rue scheme aims to overcome?

The inference that the names on the list of medical and surgical specialists in the scheme savours of advertising and touting for patients is a very lame hypothesis. If it were so, then I condemn in the same category the whole of the profession, medical or surgical, who are attached to our voluntary hospitals, and who furnish details of the appointments held for publication in the Medical Directory. I disagree entirely with the writer that inclusion in the list of a firm's advisers is equal to touting for patients. Is it not the general custom and practice of the Insurance Societies to "list" their medical advisers and referees, who must be consulted by would-be entrants to secure a clean bill of health? The eminent toxicologist giving evidence in a murder trial is stated in the Press to be the Home Office expert, and attached to this and that hospital. Does the writer suggest that because the Press publishes his name and what he is and where he attends that it is a form of advertisement?

The scheme is for the mutual benefit of employer and employee. To the employer, in providing a nursing home, benefit is very patent in that there is no waiting period, such as is usually experienced in hospital life. The patient is speedily admitted, relieved, and returns to work much sooner than he would if he were on the waiting-list of a hospital.

The employee is benefited by the speedy return to home, work, and wages, and—what is most grateful and comforting—he has not had to sponge on public charity.

It appears that the firm's medical adviser—this I take to mean the doctor in the factory—instructs which physician or surgeon on the list the patient is to see. Is this not characteristic of the lines of a general hospital? The Medical or Surgical Registrar weeds out the cases presenting themselves for examination and treatment and diverts them to the specialist whom he thinks should take charge of the case. If the De La Rue method is wrong, then our system is wrong; I suggest, however, that our system is correct, and that Messrs. De La Rue are simply following our method.

Again, who formulates the list of staff attached to a new hospital? Surely the board of management, in conjunction with advice from some medical and surgical authority. Who formulated the De La Rue list? Surely the directors, in conjunction with their medical adviser.

The position of this firm is very similar to that of a charitable institution. They have provided a nursing home, with staff attached; likewise the board of management have provided a hospital, with staff attached. In the case of a patient attending the hospital the choice of doctor does not rest with the patient. Why should it in the other case?

I firmly believe that if the very large employers of labour in London were to adopt the same principle the hospitals then would be able to treat that class of patient they originally set out to treat, assistance would soon be forthcoming to enable the hospitals to treat these "poor sufferers," and the loss of many lives would be averted through the diminution of the waiting-lists.

I hold no brief for Messrs. De La Rue & Co.—they don't even subscribe to my hospital—but the innuendoes cast upon such a praiseworthy object—an effort which has a tendency to ameliorate suffering in that it lessens, even in a small degree, the hordes of waiting cases at hospitals—is as unworthy as it is unjust.

If the De La Rue scheme is a means of illegitimate advertisement of certain selected medical men, then I must ponder, and very diligently too, the question as to whether I, by furnishing the list of staff attached to our hospital for publication, am not creating the impression that our staff is more competent than those of any similar hospital.

Yours truly,

X².

July 19, 1920.

